# Donor Nephrectomy Through Mini-Flank Incision: A Single-Centre Experience Among Nigerian Patients

**DOI:** 10.7759/cureus.25206

**Published:** 2022-05-22

**Authors:** Martin C Igbokwe, Olalekan O Olatise, Stephen O Asaolu, Abayomi A Aremu, Sadiq Abu, Uzodinma Onwuasoanya, Adefola R Adetunbi, Sani Alhassan

**Affiliations:** 1 Department of Surgery/Urology Unit, Zenith Medical and Kidney Centre, Abuja, NGA; 2 Department of Medicine/Nephrology Unit, Zenith Medical and Kidney Centre, Abuja, NGA; 3 Department of Clinical Research, Zenith Medical and Kidney Centre, Abuja, NGA; 4 Department of Surgery, Zenith Medical and Kidney Centre, Abuja, NGA; 5 Department of Surgery/Urology Unit, Aminu Kano Teaching Hospital, Kano, NGA

**Keywords:** urology, nigeria, laparoscopy, mini incision, donor nephrectomy, renal transplantation

## Abstract

Background and objective

The field of kidney transplantation in sub-Saharan Africa is still in the rudimentary stages. The majority of patients with chronic kidney disease have no access to renal replacement therapy, leading to very high mortality rates. Donor nephrectomy (DN) is an important aspect of kidney transplantation. Over the last two decades, open DN (ODN) has given way to minimally invasive techniques like laparoscopic DN (LDN) and robotic-assisted DN. In this study, we aimed to describe our experience with mini-flank incision donor nephrectomy (MIDN) at a Nigerian renal transplant center.

Materials and methods

We conducted a retrospective review of all DN cases performed at a single Nigerian kidney transplantation center over a three-year period. Information obtained from these patients was classified into pre-, intra-, and postoperative. The data included sociodemographic characteristics, preoperative preparation, details of intraoperative techniques, and postoperative findings. These were entered into a proforma and analyzed using SPSS Statistics version 21 (IBM Corp., Armonk, NY).

Results

A total of 230 patients underwent ODN during the study period. The majority of the donors were males (92.8%) with a mean age of 30.83 ±8.43 years. The body mass index (BMI) of most (76.1%) of the donors was within the normal range (18.5-24.9 kg/m^2^). The duration of DNs ranged from 72 to 154 minutes with a mean duration of 130 ±28 minutes. The length of flank incisions ranged from 7.8 to 12 cm with a mean incision length of 10.8 ±1.0 cm. Donors who had MIDN attained satisfactory postoperative pain control with about 90% of them having a BMI of <30 kg/m^2^. Oral intake and ambulation were commenced on the first postoperative day, and the cosmetic outcomes were deemed acceptable in over 90% of kidney donors.

Conclusion

Mini-incision for DN through the flank approach is a suitable alternative to LDN in the developing world where facilities and skills for LDN or robotic nephrectomies are largely unavailable. It offers a short recovery time, early ambulation, and excellent allograft outcomes.

## Introduction

Renal transplantation is the superior modality for managing end-stage renal disease and offers the patient better outcomes than dialysis [1-3]. Living-donor kidney transplants offer significant advantages over deceased donor renal allografts and are hence a suitable alternative to the latter [4]. Some of the advantages of living-donor kidney transplants include shorter duration of hemodialysis, lower chances of delayed graft function, and better one-year survival, among others [5,6]. In sub-Saharan Africa, living donors make up 100% of the donor pool for kidney transplantation as there is no system in place for a deceased donor program at the moment.

Donor nephrectomy (DN) is a key surgical procedure, where a kidney is retrieved from a donor and utilized for kidney transplantation. Over the last few decades, techniques for living-donor nephrectomy have been upgraded from the more invasive traditional open techniques to mini-incision donor nephrectomy (MIDN) and ultimately to minimal-access techniques like laparoscopic and robotic techniques [7,8]. These more modern minimal-access techniques have been shown to offer several advantages including less blood loss, quicker patient recovery, less postoperative pain, shorter hospital stay, and better cosmetic outcomes while not negatively affecting allograft function in any significant way [7,9,10].

The significantly higher cost of providing minimal-access facilities for laparoscopic and robotic surgeries has restricted the use of minimal-access nephrectomies in Nigeria and other sub-Saharan African countries [3,11]. Currently, robotic surgery is not offered in any hospital in sub-Saharan Africa, including Nigeria.

MIDN is defined as a process involving skin incisions of less than 12 cm for DN and has been performed via various approaches, including the anterior vertical and flank approaches, successfully [6,12]. Though there is a lack of consensus on the site or length of the mini-incision for DNs, many believe that the definition of the procedure is inclusive of the fact that incisions of less than 12 cm are preferred [13]. These incisions produce a better cosmetically acceptable scar, early patient recovery and discharge, and fewer postoperative complications [6,8]. In fact, some studies have found this method to be a suitable alternative to laparoscopic DN (LDN) [4]. The success of DN surgery through a mini-incision will depend on factors including the patients’ body habitus, body mass index (BMI), and the experience and competence of the surgical team [4].

This study was a retrospective analysis of all patients who underwent DN via a flank mini-incision over a three-year period at a single Nigerian kidney transplant center.

## Materials and methods

This was a retrospective review of all donors who underwent open DN (ODN) using a mini-incision between January 1, 2019, and December 31, 2021. All donors had preoperative consultation with the Urologist and had been investigated to confirm compatibility with their respective potential recipients. Investigations such as complete blood count, kidney function test, blood sugar check, liver function test, serology tests [including human immunodeficiency virus (HIV), hepatitis B and C], electrocardiography, echocardiogram, abdominopelvic ultrasound scan, and CT renal angiogram were performed for all donors to confirm their fitness for surgery and to decide on the side of DN. The side of DN was selected mostly based on the vascular anatomy with preference given to the side with fewer arteries and/or veins.

Instruments and data collection

A proforma was filled for every patient who underwent DN. The data collected included sociodemographic characteristics, BMI, side of DN, length of incision, duration of surgery, intraoperative complications, ischemic time (warm and cold), and postoperative pain. Data were also collected about the number of days admitted, analgesic requirements, duration of ileus, the commencement of oral intake, and the nature of the scar. The length of the incision was measured with a specific ruler postoperatively. Assessment of postoperative pain was done using the visual analog scale (VAS) by an independent member of the surgical team daily for the first three days and on the seventh day. Donors were discharged when they were pain-free and able to carry out basic daily routines unassisted. The wound and scar were evaluated by an independent resident surgeon.

Data were collected from the electronic medical records of patients who underwent DN during the study period. Information obtained from these patients was classified into pre-, intra-, and postoperative. The unit protocol for kidney donors in the facility was followed for all patients. Follow-up clinic visits were scheduled at one week and subsequently by telephone at one, three, and six months postoperatively.

Exclusion criteria

DN patients whose surgical incisions were >12 cm were excluded from the study.

Surgical technique for mini-flank donor nephrectomy (hospital protocol)

Procedure

The surgeries were led by two surgeons. Under general anesthesia, the patient was placed in a lateral decubitus position with the bed maximally flexed to give the “inverted V” sign. There was also a 30-degree lateral tilt of the table towards the surgeon to give the patient a diagonal lie (this maneuver prevents the renal hilum from being deep and eases surgical access to the hilum). An 8-12-cm incision was made anterior to the tip of the left 11th rib on the left side or over the anterior half of the right 11th rib and deepened through the subcutaneous tissues. The external oblique muscle, internal oblique muscle, and transversus abdominis were cut to gain access to the retroperitoneum. These muscles were undercut beyond the margins of the skin incision to ease retraction and access to the kidney. The ureter was identified and mobilized ensuring the peri-ureteric fat was not stripped off. The Gerota’s fascia was opened and the kidney mobilized with its capsule intact. 

**Figure 1:**
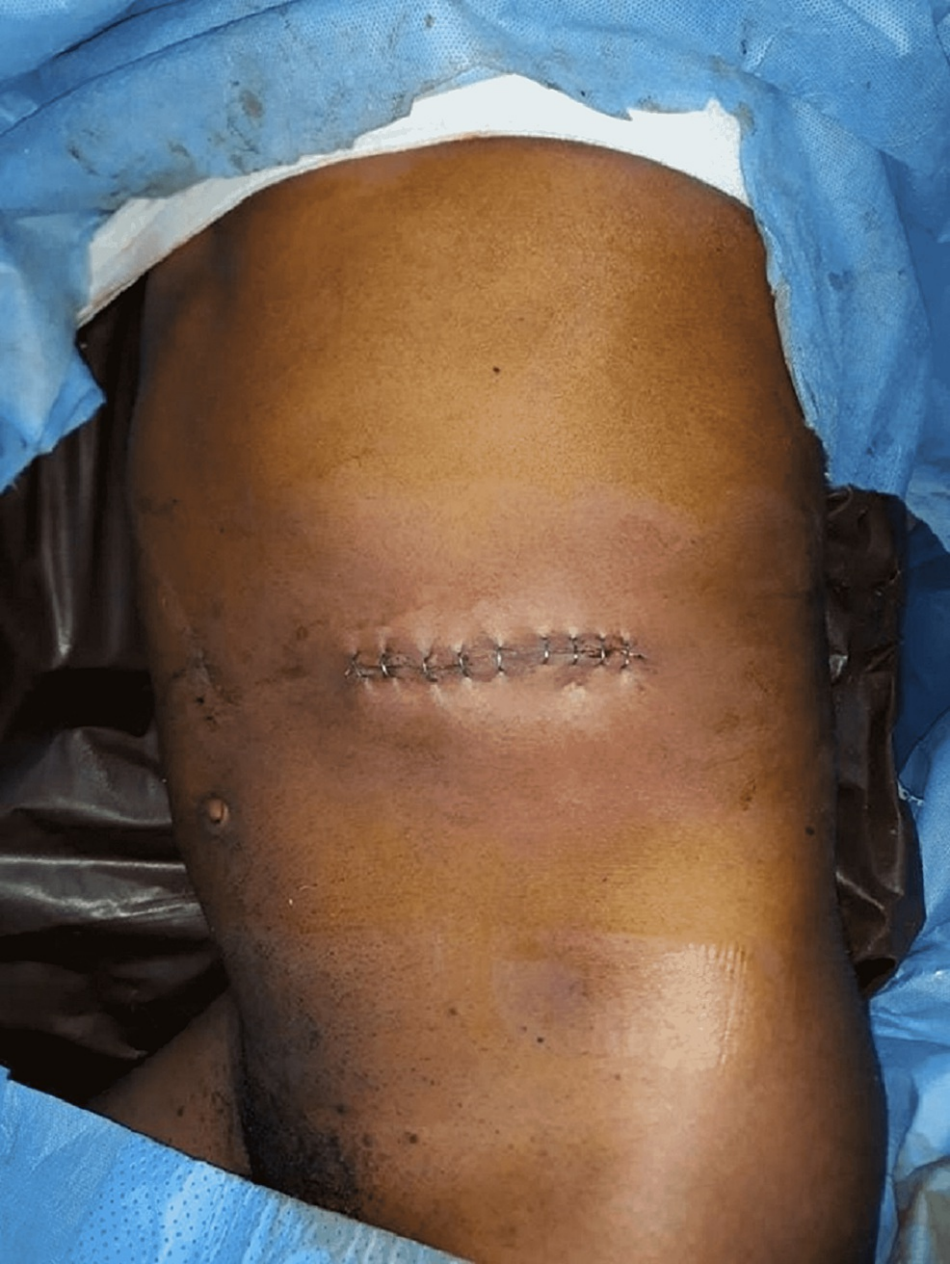
Immediate postoperative mini-incision wound

Two or three Deaver retractors were used for retraction of the peritoneum by fit surgical assistants to ensure the success of this operation and technique. Following the dissection of the renal artery and vein, the allograft was retrieved and the wound closed in layers (Figure 1).

Preoperatively, patients were admitted 24 hours before surgery and placed on nil per oral from 9 pm onwards on the day before surgery. Intravenous access was established and dextrose-containing fluid was commenced for all potential donors on the morning of surgery. Daily doses of postoperative pain medication with various opioids, non-opioid, and non-steroidal anti-inflammatory drugs (NSAIDs) were documented as daily applied total doses in milligrams or grams from the charts and analgesia protocols. There are no fixed protocols for analgesic management.

Intraoperatively, details of the side of nephrectomy, length of surgical incisions (measured with a calibrated ruler in centimeters), operation time, and ischemic times were recorded meticulously on the theatre board by the circulating scrub nurse on duty.

**Figure 2:**
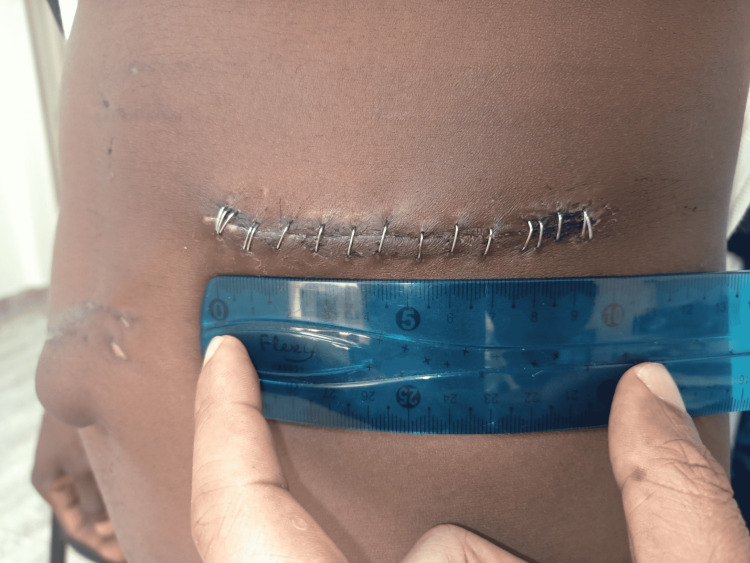
Mini-incision wound on postoperative day 3

**Figure 3:**
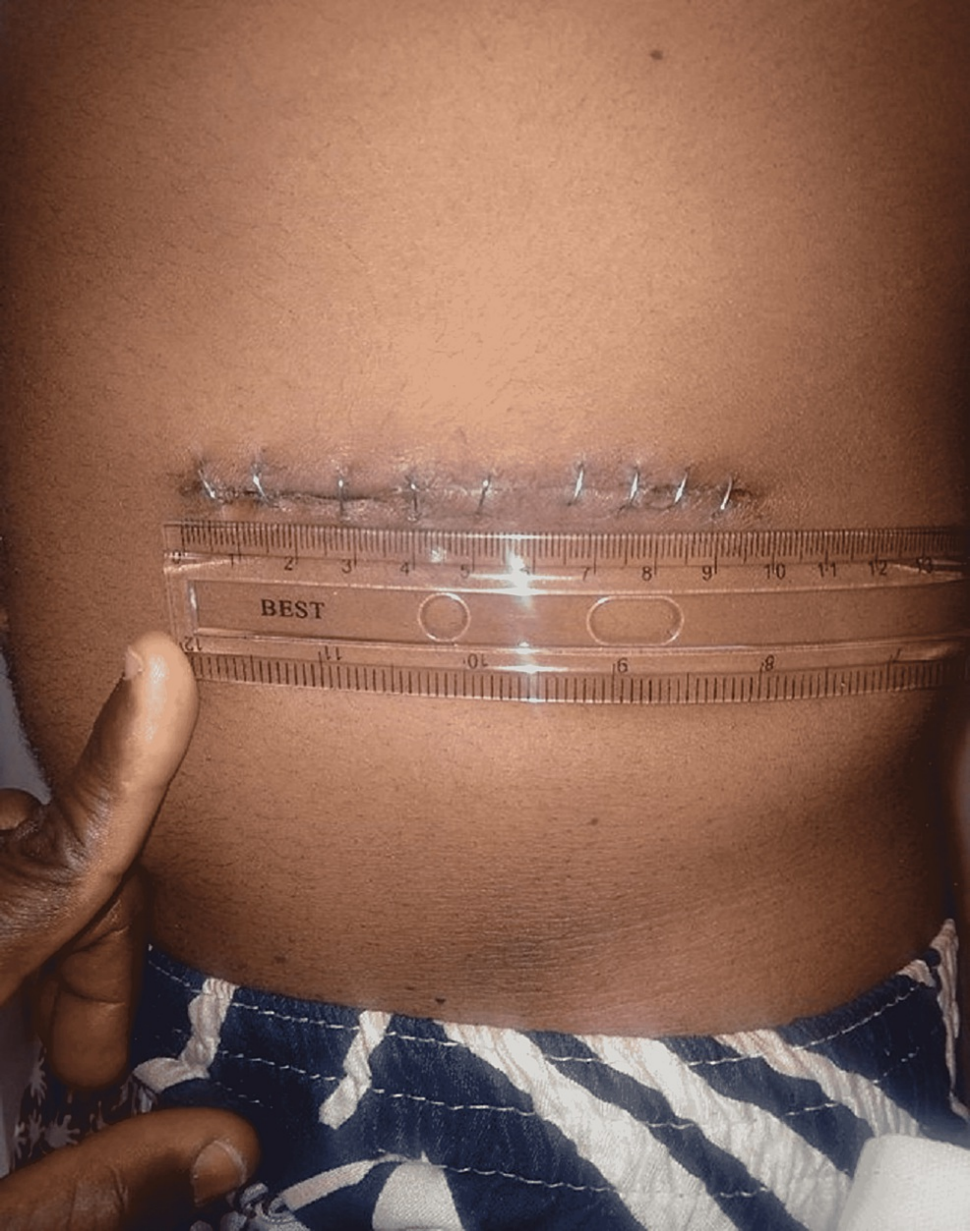
Mini-incision wound on postoperative day 7

Postoperatively, analgesic requirement and duration, duration of hospital stay, duration till oral intake, mobilization, and bowel motion were recorded. Postoperative pain was noted on days one, three, and seven (Figures 2, 3) while in the hospital using the VAS and by phone at three months.

All patients had a urethral catheter passed at surgery, which was removed on postoperative day one. Also, incentive spirometry was commenced for all patients on postoperative day one.

Data analysis

All analyses were performed using SPSS Statistics for Windows, version 22.0 (IBM Corp., Armonk, NY). Simple descriptive analysis was used to interpret the collected data. Some continuous variables were presented as mean ±standard deviation. Categorical variables were expressed as frequencies and percentages.

Ethics and approval

Consent was sought and received from every patient who was included in the study. The data collected were anonymized before entry and analysis. This study was reviewed and approved by the Federal Capital Territory Health Research Ethics Committee (FCTHREC) with approval number FHREC/2021/01/146/14-12-2021.

## Results

A total of 230 patients had DN using a mini-incision during the period reviewed. The majority of the donors were males (92.8%) with a mean age of 30.83 ±8.43 years (Table 1).

**Table 1:** Sociodemographic and some clinical characteristics of the donors (n=230) SD: standard deviation

Variable		Frequency (n)	Percentage (%)
Age (years)	<20	5	2.0
	21–30	147	63.8
	31–40	62	27.3
	41–50	15	6.6
	51–60	1	0.3
	Mean ±SD	30.83 ±8.43	
	Range (min, max)	34 (19, 53)	
Sex	Male	214	92.8
	Female	16	7.2
Body mass index (Kg/m^2^)	<20	5	2.2
	20–24.9	175	76.1
	25–29.9	37	16
	30–34.9	8	3.5
	35–39.9	5	2.2
Complications	Excessive primary hemorrhage	12	5.2
	Pleural injury	5	2.2
	Peritoneal breach	20	8.7
	Surgical site infection	6	2.6
	Suboptimal graft function	5	2.2
	Perioperative mortality	0	0

The BMI of most (76.1%) of the donors was within the normal range (18.5-24.9 kg/m^2^). These 230 patients accounted for 75.6% of all DN surgeries performed in the three-year study period. The duration of DNs ranged from 72 to 154 minutes with a mean duration of 130 ±28 minutes. The length of flank incisions ranged from 7.8 to 12 cm with a mean incision length of 10.8 ±1.0 cm (Table 2).

**Table 2:** Perioperative characteristics of the donors (n=230) SD: standard deviation

Variable		Frequency (n)	Percentage (%)
First warm ischemic time (seconds)	Mean ±SD	106.41 ±42.50	
	Median	110.00	
	Range (min, max)	480 (25, 505)	
Cold ischemic time (minutes)	Mean ±SD	33.26 ±20.01	
	Median	39.00	
	Range (min, max)	62 (10, 72)	
Second warm ischemic time (minutes)	Mean ± SD	40.05 ±13.81	
	Median	49.00	
	Range (min, max)	64 (18, 82)	
Side of nephrectomy	Right	66	28.9
	Left	164	71.1
Length of incisions (cm)	8–10	42	18.3
	10–12	188	81.7
	Mean ±SD	10.8 ±1.0	
	Range (min, max)	4.2 (7.8, 12)	

The significant intraoperative complications encountered were excessive primary hemorrhage (5.2%) and peritoneal breach (8.7%). Postoperative pain control was deemed satisfactory with a pain score of 4 or less in 204 patients (88.6%). Higher pain scores and higher analgesic requirements were observed in patients with a BMI >30 kg/m^2^ (p=0.02) and following rib resection (p=0.001).

Oral intake and ambulation out of bed were established on postoperative day one in all patients. The mean duration to bowel motion (feces) from surgery was 2.3 ±1.5 days; 221 (96%) of donors were discharged on the third day postoperatively (fourth day of admission) with a satisfactory outcome. Of note, 207 (90.1%) of these patients were satisfied with their wound scars and the surgical experience one month after surgery. However, 41 (17.8%) and 23 (10%) of the donors complained of some pain at the operation site one week and one month following the surgery respectively. However, this pain was not significant enough to affect their return to work and other daily routines.

Suboptimal graft function in the immediate postoperative period due to acute tubular necrosis or delayed graft function was found in five (2.2%) patients. However, these grafts satisfactorily resolved and the patients achieved optimal function within the first two to three weeks of surgery.

## Discussion

Kidney transplantation remains the gold standard for renal replacement therapy worldwide [3]. DN is key to procuring a healthy renal allograft. Over the years, safer and less invasive techniques for DN have been employed with the aim of improving donor outcomes including shorter length of incision, minimal postoperative pain, reduced duration of hospital stay, and early ambulation bowel motion among others [14]. Some earlier studies had shown poor patient acceptance of ODN due to increases postoperative pain and poor cosmetic results pertaining to scars when compared to LDN [15].

The majority of our study population were young males in their third and fourth decades of life, which contrasts with several other studies where more female donors were encountered with a mean age of over 50 years [16,17]. This is likely due to the fact that most Africans believe that men are stronger than their female counterparts, and hence able to withstand the rigors of surgery [18]. In our center, DNs were performed 2.5 times more on the left than on the right side; this finding is similar to reports in several other international studies [4,16]. This is commonly attributed to the longer left renal vein, which makes anastomosis in the recipient less laborious.

Although LDN has become the gold standard worldwide, the use of mini-incisions has been accepted as an alternative in developing countries where facilities and skills for laparoscopy are still rudimentary [14]. In this study, 188 patients (81.7%) had DNs using incisions between 10 and 12 cm, which is similar to the study by Kok et al. [4], and this is significantly smaller than the regular flank incisions that are 20-30 cm long as described by Vernadakis et al. in Greece [17]. The BMI of most of our donors was within the normal range. Mini-incisions have been reported to be easier when performed in such patients compared to overweight and obese patients who end up with larger skin incisions and may otherwise benefit from LDN [13,17].

The duration of the first warm ischemic time was found to be slightly shorter than that described in the literature for LDN [4,17], while the total operation time was much shorter when compared to other studies where both ODN (157 minutes) and LDN (240 minutes) were performed [4]. Intraoperative complications were very few in this study, signifying that MIDN is quite safe in expert hands, which is evidenced by the fact that only two patients required re-exploration for significant postoperative hemorrhage.

The majority of the patients in this study commenced oral intake and were ambulated within 24 hours of surgery, which aligns with the findings of an Indian comparative study by Sinha et al. [19]. In comparison, the ODN arm of this study commenced oral intake and ambulation on day three. Also, the LDN patients were discharged home on day five after surgery, which is significantly later than the MIDN patients in this series who were discharged on day three. This could be attributed to the high proportion of young male patients in our cohort with a high-performance status as compared to other studies with older patients, a larger female population, and possible marginal donors [17].

Yadav et al. found that LDN patients had a longer hospital stay and warm ischemia time, as well as higher operative and postoperative costs [14], suggesting that some of the perceived advantages of LDN may need to be reviewed. This study also showed a low rate of suboptimal graft function (2.2%) in the immediate postoperative period, which is similar to the findings from other studies [7,19].

## Conclusions

Based on our findings, mini-incision for DN through the flank approach is a suitable alternative to LDN in the developing world where facilities and skills for laparoscopic or robotic nephrectomies are often unavailable. This technique offers a shorter operative time and first warm ischemic time with minimal morbidities to the patient. However, It is not suitable for overweight and obese patients who will require larger incisions for adequate access.
